# Comparing Transvaginal Ultrasound Measurements of Cervical Length to Bishop Score in Predicting Cesarean Section Following Induction of Labor: A Prospective Observational Study

**DOI:** 10.7759/cureus.54335

**Published:** 2024-02-16

**Authors:** Parul Sinha, Mansi Gupta, Snehlata Meena

**Affiliations:** 1 Department of Obstetrics and Gynecology, All India Institute of Medical Sciences, Raebareli, Raebareli, IND; 2 Department of Obstetrics and Gynecology, Eras Lucknow Medical College and Hospital, Lucknow, IND

**Keywords:** vaginal delivery, cesarean section, transvaginal ultrasonography cervical length, induction of labour, bishop score

## Abstract

Introduction: Bishop score (BS) has been used to see the favorability of the cervix for induction of labor (IOL), but it has limitations in today's diverse patient population. We aimed to assess the predictive value of transvaginal ultrasound (TVUS) measurements of cervical length (CL) compared to BS in determining the likelihood of cesarean section (CS) following IOL.

Methodology: A prospective observational study was conducted on 120 women requiring IOL in a tertiary care hospital in central India. The inclusion criteria of the study were antenatal women more than 18 years of age, in need of IOL, having a singleton pregnancy with a gestational age of > 37 weeks as determined from the date of the last menstrual period and confirmed by sonographic measurements in the first trimester, presenting with a cephalic presentation, and having intact fetal membranes. Women with prior uterine scars and those unwilling to IOL were excluded from the study. TVUS was done just before induction. Statistical analyses were done to compare the predictive abilities of CL and BS for CS.

Results: The mean age and gestation period were 25.96 years and 39 weeks 3 days, respectively. The majority of the study population comprised multigravida (69, 57.5%), followed by primigravida (47, 39.2%), and grand multigravida (≥ G5) (4, 3.3%). Post-maturity (34, 28.3%), preeclampsia (21, 17.5%), and intrahepatic cholestasis of pregnancy (17, 14.2%) were common indications for induction. The overall CS rate was 35.8% (43/120). Women with CS had lower BS (3.60 vs. 4.70, *P *= 0.010) and higher CL (31.5 mm vs. 23.4 mm, *P *< 0.001). CL exhibited an area under the curve (AUC) of 0.857, outperforming BS (AUC = 0.643) in predicting CS. Using a CL cutoff of 26.5 mm yielded sensitivity (79.1%), specificity (81.8%), and overall accuracy (80.8%).

Conclusions: TVUS measurement of CL (>26.5 mm) demonstrated superior predictive ability for CS following labor induction compared to BS (≤5). This study highlights the potential of CL measurement as an objective and reliable tool for optimizing decision-making in labor induction.

## Introduction

Labor induction is a common obstetric procedure performed in a significant proportion of deliveries worldwide (1.4%-35%), offering potential benefits like reducing perinatal mortality, especially in post-term pregnancies [1]. Various studies have investigated factors influencing the success of labor induction, with cervical ripening playing a crucial role in facilitating vaginal birth. In 1964, Bishop introduced a cervical scoring system using manual examination to evaluate cervical ripening [2]. Research indicates that a score exceeding 8 is favorable for successful labor induction, resulting in vaginal birth for over 90% of women [3-5]. However, it has multiple shortcomings as compared to current practice. Bishop's original scoring system for induced labor, developed nearly 60 years ago, focused on achieving vaginal delivery (VD) within four hours and elective induction at term for multiparous women with prior vaginal births [6]. However, modern obstetrics predominantly involves labor induction for maternal or fetal indications, often in nulliparous women and pre-term pregnancies, with outcomes evaluated within 24 hours and advanced induction methods. Despite these significant shifts, Bishop score (BS) is widely used, lacking empirical evidence for its effectiveness in today's diverse and medically complex patient population [6]. The increased utilization of BS in obstetrics may be attributed to recent developments in the ARRIVE trial [7]. This extensive, well-structured, randomized trial demonstrated that inducing labor at 39 weeks reduces the likelihood of cesarean delivery when compared to an expectant approach [7]. Furthermore, contemporary methods of labor induction differ significantly from those employed during Bishop's era, which predominantly involved oxytocin and amniotomy. Nowadays, there is a range of new pharmacological agents and mechanical techniques designed to imitate the cervical changes believed to precede natural labor [6]. While BS continues to be widely utilized, it is marred by substantial inter- and intra-observer variability. In a study using polyvinyl chloride pipes to simulate cervical examinations, 56.3% accuracy was achieved in determining exact diameters, improving to 89.5% when allowing a ±1 cm error [8]. Interobserver variability was initially over 50%, but it decreased to 10.5% when the same error margin was permitted, highlighting the precision of cervical diameter measurements with a margin of +/- 1 cm and the significance of intra-observer variability in assessing dysfunctional labor [8]. Moreover, evaluating specific cervical changes, such as funneling at the internal os and cervical length (CL), can be challenging when the cervical os is closed. Consequently, efforts have been made to explore alternative, more objective methods for predicting the success of labor induction. Therefore, this study was conducted to assess the relationship between transvaginal ultrasound (TVUS) measurements of the cervix and BS in predicting the success of labor induction. The study hypothesized that TVUS measurements of CL would serve as a highly sensitive method for predicting the outcome of labor induction. This could potentially enhance the decision-making process for labor induction, either by supplementing the existing BS or potentially serving as an alternative, more reliable gold standard for pre-induction cervical evaluation.

## Materials and methods

This was a prospective observational study which was conducted in the Obstetrics and Gynecology Department of a tertiary care hospital located in central India. It focused on women who required medically indicated induction of labor (IOL). The study was conducted over 18 months, from February 2017 to August 2018. The inclusion criteria of the study were antenatal women more than 18 years of age, in need of IOL, having a singleton pregnancy with a gestational age of >37 weeks as determined from the date of the last menstrual period and confirmed by sonographic measurements in the first trimester, presenting with a cephalic presentation, and having intact fetal membranes. Women with prior uterine scars and those unwilling to IOL were excluded from the study. TVUS was done just before induction.

The sample size was calculated based on a previous study done by Park et al., which found that the proportion of women undergoing VD was 78.8% (127/161) [9]. In this study, we determined the sample size based on specified parameters, including a population proportion of 0.78 (representing 78% proportion of induction in the group having BS ≤ 5), a type 1 error rate of 0.05 (5%), an acceptable margin of error of 0.10 (10%), a desired study power of 0.90 (90%), and a data loss factor of 0.10 (10%). The calculated sample size for the study was found to be 120.

The study was started after getting approval from the Institutional Ethics Committee (approval number: ELMC/R_cell/EC/2017/64). The participants upon arriving at the labor ward provided written informed consent and underwent a series of assessments, including history-taking, clinical examinations, and obstetric examinations. A vaginal examination was performed to assess the status of the uterine cervix and calculate the BS by a single experienced obstetrician in all the cases. A TVUS was performed by a single experienced clinician in all the cases following standard techniques, as described by Kagan and Sonek to measure CL using specific equipment (Elpro GE LOGIQ P5 USG machine with 8 MHz transducer probe) [10]. All women were positioned in dorsal lithotomy. Before the TVUS scan, patients were required to empty their bladders. The cervix was examined longitudinally, identifying the cervical canal and surrounding mucosa. Care was taken to minimize pressure on the cervix from the probe. CL was measured using calipers at both the external and internal os, and a repeat measurement was conducted for confirmation. These procedures typically lasted three to four minutes.

Patients with a BS of 5 or less were given tablet misoprostol vaginally, while those with a BS of 6 or higher were given intravenous oxytocin infusion. The misoprostol was given at a dose of 25 microg every four hours per vaginally, with a maximum of eight doses or until the cervix became clinically favorable or the patient started having good uterine contractions. Oxytocin infusion was administered with 2.5 units of oxytocin (Syntocinon) in 500 mL of Ringer's lactate solution. The infusion rate was initially set at 2.5 mU/minute and increased by 2.5 mU/minute every 30 minutes, up to a maximum of 20 mU/minute. All women were closely monitored until delivery, with the primary outcome being the occurrence of a CS. Misoprostol was selected over Dinoprostone gel for IOL in this study due to its stability at room temperature, ease of administration, and lower cost. At least 12 hours of oxytocin and rupture of membranes in nulliparous and 15 hours in multiparous women were defined as failure of induction [11].

Maternal monitoring

Temperature, pulse, and blood pressure were monitored at least every four hours. If membranes were ruptured for many hours or if there was borderline temperature elevation, the temperature was checked hourly.

Fetal monitoring

In the absence of any abnormalities, the fetal heart rate was monitored immediately after a contraction at least every 30 minutes and then every 15 minutes during the second stage.

Data were collected and tabulated in an Excel sheet. The collected data were analyzed using IBM SPSS Statistics for Windows, Version 21.0. (IBM Corp., Armonk, NY). Descriptive statistics of the quantitative data were described as mean, standard deviation, and range, and those of the nominal data were described as frequency and percentage. The normality of the distribution of the data was tested using the Shapiro-Wilk Test. The independent samples t-test was used for normally distributed data and the Mann-Whitney U test was used for non-normally distributed data. Age, BS, and CL were non-normally distributed, while the period of gestation was normally distributed. Group differences between categorical data were analyzed using the chi-square test. The predictive ability of continuous variables for the dichotomized outcome variable was tested using receiver operating characteristic (ROC) curve analysis. *P*-value < 0.05 was considered statistically significant.

## Results

In this prospective study, we aimed to evaluate and compare the predictive values of transvaginal ultrasonographic assessments of CL and BS for determining the likelihood of CS as an outcome of labor induction (Table 1).

**Table 1:** Components of Bishop score.

Components	Scores			
	0	1	2	3
Dilatation (cm)	0	1-2	3-4	≥5
Effacement (%)	0-30	40-50	60-70	≥80
Station	-3	-2	-1 to 0	+1, +2
Consistency	Firm	Medium	Soft	-
Position	Posterior	Mid position	Anterior	-

A total of 120 pregnant women, meeting the inclusion criteria, were enrolled in the study. The age distribution of the participants ranged from 20 to 35 years, with the majority falling in the 20-25 years age group (68, 56.7%), followed by 26-30 years (40, 33.3%), and 31-35 years (12, 10%). The mean age of the participants was 25.96 ± 3.56 years. Multigravida (69, 57.5%) constituted the majority of the study population followed by primigravida (47, 39.2%) and grand multigravida (≥ G5) (4, 3.3%). The gestational age at enrollment varied between 37 weeks 1 day and 42 weeks 2 days, with the majority (78, 65%) having a gestational age of less than 40 weeks, while the remaining cases (42, 35%) had a gestational age exceeding 40 weeks. The mean period of gestation (POG) was 39 weeks 3 days (range 37 weeks 1 day to 42 weeks 2 days) (Table 2).

**Table 2:** Baseline characteristics and outcomes of participants in the study (n = 120). ^a^Mild placental abruption (no sign of maternal/fetal distress). TVS, transvaginal sonography; CS, cesarean section; VD, vaginal delivery

Characteristics	Values
Age (Years)	25.96 ± 3.56 (20-35)
Bishop score	5.55 ± 2.03 (0-9)
Cervical length (mm)	26.3 ± 6.23 (15-44)
Age group-wise distribution, *n* (%)	
20-25 years	68 (56.7%)
26-30 years	40 (33.3%)
31-35 years	12 (10.0%)
Gravida status, *n* (%)	
G1	47 (39.2%)
G2	34 (28.3%)
G3	23 (19.2%)
G4 or above	16 (13.3%)
Duration of pregnancy, *n* (%)	
≤40 weeks	78 (65%)
>40 weeks	42 (35%)
Period of gestation (weeks)	39.3 ± 1.1 (37-42.2)
Distribution of cervical factors	
Cervical dilatation, *n* (%)	
Closed	13 (10.8%)
1-2 cm	81 (67.5%)
3-4 cm	26 (21.7%)
Effacement, *n* (%)	
0%-30%	75 (62.5%)
40%-60%	37 (30.8%)
60%-80%	8 (6.7%)
Position, *n* (%)	
Posterior	33 (27.5%)
Mid position	50 (41.7%)
Anterior	37 (30.8%)
Consistency, *n* (%)	
Firm	18 (15%)
Medium	27 (22.5%)
Soft	75 (62.5%)
Station, *n* (%)	
-3	95 (79.2%)
-2	24 (20%)
-1	1 (0.8%)
Distribution of cases according to Bishop score, *n* (%)	
<4	18 (15.0%)
4-6	67 (55.8%)
>6	35 (29.2%)
Distribution of cases according to TVS measurement of cervical length	
≤25 mm	62 (51.7%)
25.1-30 mm	30 (25%)
>30 mm	28 (23.3%)
Indications for Induction of labor, *n* (%)	
Postmaturity	34 (28.3%)
Preeclampsia	21 (17.5%)
Intra-hepatic cholestasis of pregnancy	17 (14.2%)
Oligohydramnios	13 (10.8%)
Fetal growth restriction	10 (8.3%)
Intra-uterine demise	6 (5%)
Rh-negative status with isoimmunization	6 (5%)
Antepartum hemorrhage^a^	5 (4.2%)
Maternal request	4 (3.3%)
Gestational diabetes mellitus	3 (2.5%)
Polyhydramnios	1 (0.8%)
Method of induction, *n* (%)	
Misoprostol	79 (65.8%)
Oxytocin	41 (34.2%)
Mode of delivery, *n* (%)	
CS	43 (35.8%)
VD	77 (64.2%)

Cervical assessments were performed through vaginal examinations, evaluating parameters such as dilatation, effacement, consistency, and the position and station of the fetal head to calculate the BS. The majority of women (81, 67.5%) exhibited cervical dilation of 1-2 cm, followed by 3-4 cm (26, 21.7%). A smaller portion (13, 10.8%) had a closed cervix. Effacement was observed in the range of 0%-30% for 75 (62.5%) women, 40%-60% for 37 (30.8%) women, and 60%-80% for 8 (6.7%) women, with none displaying effacement exceeding 80%. The majority (75, 62.5%) had a soft cervical consistency, 27 (22.5%) women had a medium consistency, and 18 (15%) women had a firm consistency. Cervical positioning was predominantly mid-positioned (50, 41.7%), followed by anterior (37, 30.8%) and posterior (33, 27.5%) positioning. The fetal head station was most frequently identified as -3 (95, 79.2%), followed by -2 (24, 20.0%) and -1 (1, 0.8%), with no cases showing a station value of +1 or +2. Calculated BS ranged from 0 to 9, with the majority falling in the 4-6 range (67, 55.8%), followed by scores >6 (35, 29.2%). A total of 18 (15%) cases had a BS < 4, and the mean BS was 5.55 ± 2.03 (Table 2). CL measurements ranged from 15 to 44 mm, with the majority (62, 51.7%) having a CL of < 25 mm, followed by 25.1-30 mm (30, 25%) and >30 mm (28, 23.3%). The mean CL was 26.3 ± 6.23 mm (Table 2). Post-maturity was the most common indication for induction (34, 28.3%), followed by preeclampsia (21, 17.5%), intrahepatic cholestasis of pregnancy (IHCP, 17, 14.2%), oligohydramnios (13, 10.8%), and fetal growth restriction (10, 8.3%). Less common indications included intrauterine demise (6, 5%), Rh-negative status with isoimmunization (6, 5%), antepartum hemorrhage due to mild placental abruption (no sign of maternal/fetal distress) (5, 4.2%), maternal request (5, 4.2%), gestational diabetes mellitus (3, 2.5%), and polyhydramnios (1, 0.8%). Misoprostol was utilized for induction in 79 (65.8%) cases, while oxytocin was used in 41 (34.2%) cases. Ultimately, the outcome of full-term normal delivery was achieved in 77 (64.2%) cases, while 43 (35.8%) cases resulted in CS (Table 2).

The age distribution was similar among the women undergoing CS and VD, with mean ages of 25.44 ± 3.27 years and 26.25 ± 3.71 years, respectively (*P* = 0.215). BS was lower (3.60 ± 2.31 vs. 4.70 ± 2.15, *P *= 0.009), and CL was higher (31.5 ± 2.31 mm vs. 23.4 ± 3.62 mm, *P *< 0.001) in the CS group compared to the VD group. The proportion of primigravida was significantly higher in the CS compared to that in VD (23/43, 53.5%, vs. 24/77, 31.2%, *P *= 0.016). However, there was no difference between the period of gestation (39.2 ± 1.01 vs. 39.3 ± 1.16 weeks, *P* = 0.811) and the method of induction among the two groups (Table 3).

**Table 3:** Distribution of various variables in the cesarean section (CS) and vaginal delivery (VD) groups (n = 120). ^a^Mann-Whitney U test. ^b^Independent samples test. ^c^Chi-square test.

	Cesarean section (*n *= 43)	Vaginal delivery (*n *= 77)	*P*-value
Age (years)	25.44 ± 3.27	26.25 ± 3.71	0.215^a^
Bishop score	3.60 ± 2.31	4.70 ± 2.15	0.009^a^
Cervical length (mm)	31.5 ± 6.64	23.4 ± 3.62	<0.001^a^
Period of gestation (weeks)	39.2 ± 1.01	39.3 ± 1.16	0.811^b^
Gravida			
Primigravida	23 (53.5%)	24 (31.2%)	0.016^c^
Multigravida	20 (46.5%)	53 (68.8%)	
Duration of pregnancy			
<40 weeks	30 (69.8%)	48 (62.3%)	0.413^c^
>40 weeks	13 (30.2%)	29 (37.7%)	
Method of induction			
Misoprostol	29 (67.4%)	50 (64.9%)	0.781^c^
Oxytocin	14 (32.6%)	27 (35.1%)	

**Figure 1:**
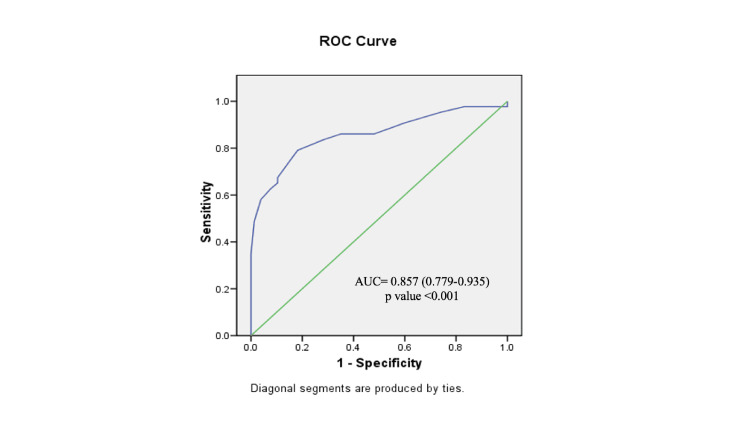
Receiver operator characteristic (ROC) curve for prediction of cesarean section with sonographically measured cervical length as a predictor. AUC, area under the curve

**Figure 2:**
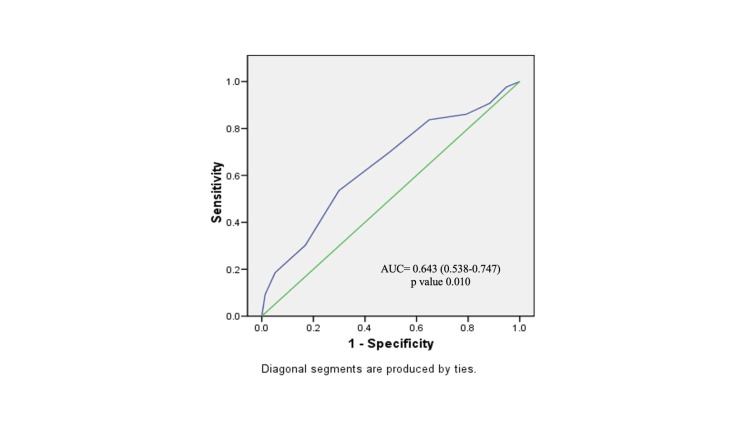
Receiver operator characteristic curve for prediction of cesarean section with Bishop score as a predictor. AUC, area under the curve

The ROC curve for CL exhibited an area under the curve (AUC) of 0.857 (95% CI = 0.779-0.935, *P* < 0.001) (Figure 1). When a CL of 26.5 mm was chosen as the cutoff, it demonstrated a sensitivity of 79.1% and a specificity of 81.8%, with positive predictive value (PPV) and negative predictive value (NPV) of 70.8% and 87.5%, respectively, and an overall accuracy of 80.8% (Table 4). In comparison, the ROC curve for the BS yielded an AUC of 0.643 (95% CI = 0.538-0.747, *P* = 0.010), which indicates that BS also predicts the likelihood of CS in IOL, although less strongly as compared to CL alone (Figure 2). Using a BS cutoff of <5, it displayed a sensitivity of 83.7% and a specificity of 35.1%, with PPV and NPV of 41.9% and 79.4%, respectively, and an overall accuracy of 52.5% (Table 4). When we compared the AUC of both ROC curves, CL emerged as the superior predictor of CS following IOL, with an AUC of 0.857 (Figure 1), as compared to the BS AUC of 0.643 (*P* < 0.001) (Figure 2). Further analysis revealed that CL was more specific (81.8% vs. 35.1%, *P* < 0.001), had a higher PPV (70.8% vs. 41.9%, *P* = 0.001), and had greater overall accuracy (80.8% vs. 52.5%, *P* < 0.001) than the BS (Table 4). However, there were no significant differences in sensitivity (79.1% vs. 83.7%, *P* = 0.579) or NPV (87.5% vs. 79.4%, *P* = 0.278) between these two predictors (Table 4).

**Table 4:** Comparison of efficacy of cervical length and Bishop score and for prediction of cesarean delivery. AUC, area under the curve; CI, confidence interval

Prediction of overall cesarean delivery			
	Cervical length >26.5 mm	Bishop score (<5)	*P*-value
AUC (95% CI)	0.857 (0.779-0.935)	0.643 (0.538-0.747)	-
*P*-value	<0.001	0.010	-
Sensitivity	79.1%	83.7%	0.579
Specificity	81.8%	35.1%	<0.001
Positive predictive value	70.8%	41.9%	0.001
Negative predictive value	87.5%	79.4%	0.278
Accuracy	80.8%	52.5%	<0.001

## Discussion

Cervical assessment through TVUS in pregnancy has been practiced since 1986, with no added risks to the mother or fetus [1]. Numerous studies have suggested that TVUS measurement of CL is a sensitive method for predicting the success of labor induction (Table 5) [1,4,9,12-29]. For instance, Daskalakis et al. found that a CL of <27 mm, as measured by TVUS, had a 76% sensitivity and 75.5% specificity for predicting VD compared to BS [16]. TVUS also appeared to be better tolerated with lower pain scores compared to digital vaginal examination [1]. However, conflicting results have emerged from various studies over the years, with some failing to show a significant correlation between cervical measurements via ultrasound and the primary outcome of successful VD. A systematic review in 2015 reported no significant difference between TVUS and BS in terms of outcomes such as vaginal birth, cesarean delivery, and induction-to-delivery interval [30].

**Table 5:** Review of literature of relevant studies comparing the predictive role of CL and BS in the outcome of IOL. ^$^TVS cervical score (this includes parameters of CL, funneling, the position of the cervix, and distance of presenting part from external os). BS, Bishop score; CL, cervical length; CSR, cesarean section rate; VD, vaginal delivery; IOL, induction of labor; BMI, body mass index; TVUS, transvaginal ultrasound; TVS, transvaginal sonography; PPV, positive predictive value; NA, not available

Author	Country	Sample size	Study type	Proportion of nulliparous women	Period of gestation (weeks)	Cesarean section rate (%)	Conclusions
Rozenberg et al. (2000) [12]	France	128	Prospective	48.4	NA	12.5	BS ≥ 6 and CL ≤ 26 mm: associated with spontaneous onset of labor within seven days
Chandra et al. (2001) [13]	Canada	122	Prospective	64	41.2	20	CL and BS do not predict successful IOL in post-term pregnancy
Yang et al. (2004) [14]	South Korea	105	Prospective	74	40.5	11	CL is a useful and independent predictor of successful IOL not BS
Roman et al. (2004) [15]	France	106	Prospective	NA	39.8	17.9	Induction failure was associated with low BS before and six hours after the start of induction, increased clinical CL, and increased ultrasound anterior cervical lip area. CL was not better than BS for IOL prediction in an unfavorable cervix.
Daskalakis et al. (2006) [16]	Greece	137	Prospective	100	40	32.8	CL is a good predictor of a successful IOL at term in nulliparous women.
Park (2007) [9]	South Korea	161	Prospective	100	39.8	21	CL ≥ 28 mm was a predictor of failed IOL and was not BS.
Keepanasseril et al. (2007) [17]	India	138	Prospective	100	38	23.2	CL and posterior cervical angle were better than BS in predicting successful IOL in nulliparous women.
Eggebø et al. (2009) [4]	Norway	275	Prospective	NA	NA	NA	Digital assessment of fetal head descent, CL, and position can be replaced with ultrasound measurements for predicting successful IOL.
Meijer-Hoogeveen et al. (2009) [18]	The Netherlands	102	Prospective	66.7	41.1	13	The maternal postural change improves the accuracy of CL for predicting VD after IOL at term.
Uyar et al. (2009) [19]	Turkey	189	Prospective	71	40.2	16.9	BMI, CL: better predictors of successful IOL compared to BS
Groeneveld et al. (2010) [20]	Belgium and the Netherlands	110	Prospective	60	39.9	17	Best cutoff value for BS: 3 CL: Not a significant independent predictor of VD within 96 hours.
Bastani et al. (2011) [21]	Iran	200	Prospective	66.7	39.6	28.5	TVUS has the potential to replace the traditional BS.
Cubal et al. (2013) [22]	Portugal	197	Prospective	61.9	40.7	15.7	BS and CL are good predictors of successful IOL in nulliparous women.
Kehila et al. (2015) [23]	Tunis	77	Prospective	100	41	33.8	CL is better than BS: in predicting cervical ripening success and VD in nulliparous women at 41 weeks of gestation with an unfavorable cervix
Bajpai et al. (2015) [24]	India	131	Prospective	70.1	NA	13.1	TVS cervical score^$^ of ≥ 4 performed better than BS.
Khazardoost et al. (2016) [25]	Iran	100	Prospective	NA	NA	43	The cutoff for CL and BS were 16 and 5 mm, respectively, predicting successful IOL.
El Mekkawi et al. (2017) [26]	Egypt	210	Prospective	100	38	23.8	CL < 28 mm was more specific with more PPV compared to modified BS in the prediction of successful IOL.
Sharma et al. (2017) [27]	India	100	Prospective	NA	NA	47	BS < 6 and CL > 3 cm: predicts cervical unfavorability. CL was a better predictor for the success of IOL than BS.
Khandelwal et al. (2018) [28]	India	62	Prospective	100	NA	16.7	BS > 4 and CL ≤ 25 mm predict successful IOL within six hours. BS is superior to CL in predicting the response to IOL.
Rizzo et al. (2020) [29]	Italy	457	Prospective	100	37	22.5	CL > 24 mm at 36 to 37 weeks predicted delivery ≥40 weeks Identified women for elective IOL at 39 weeks.
Abdullah et al. (2022) [1]	Malaysia	294	Clinical Trial	44.9	39.1	29.6	CL ≤ 27 mm and BS ≥ 4 were independent predictors of successful labor inductions. CL and BS provided similar diagnostic accuracy.

This study aimed to assess the predictive capability of BS in comparison to the CL for determining the likelihood of CS in induced labor. The mean age of the women in our study was 25.96 ± 3.56 years. In India, the third decade of life is widely considered to be the optimal time for conceiving and becoming a mother, a trend that our study aligns with, as the majority (108, 90%) of our study population falls within this age group.

In this study, the maximum number of women was Gravida 1 (47, 39.2%) followed by Gravida 2 (34, 28.3%). Bajpai et al. had 70.1% primipara in their study [24], whereas Cubal et al. and Daskalakis et al. had a 61.9% and 100% prevalence of primipara, respectively, in their studies [16,22]. That being said, when compared to other studies, this study had a higher proportion of multipara women.

In this study, all the women had a gestational age of ≥37 weeks. However, Cubal et al. reported the mean gestational age to be above 40 weeks, whereas Kehila et al. included all the women at exactly 41 weeks of pregnancy [22,23]. Bajpai et al. and Khandelwal et al. had similar gestational age distributions in their studies [24,28].

In our study, the majority of them (67, 55.8%) had BS in the range of 4-6 and a mean BS of 5.55 ± 2.03, which was relatively higher compared to findings in other studies. For example, previous research by Cubal et al. reported a majority of women with BS <5 (56.9%) [22], and Khazardoost et al. found a mean BS of 4.3 in their study population [25]. Interestingly, the proportion of women with BS greater than 6 in this study was 35 (29.2%), whereas another study by Sharma et al. found this proportion to be only 8% in their study [27]. The relatively higher BS in this study could be attributed to two potential factors: first, the study population might have had a lower proportion of women at risk for cesarean delivery, and second, the subjective nature of BS evaluation.

In this study, CL measurements were obtained through TVUSs and ranged from 15 to 44 mm, with a mean length of 26.3 ± 6.23 mm. In comparison to our findings, other studies by Bajpai et al., Khazardoost et al., and Khandelwal et al. reported slightly lower mean CL of 25.4, 25.2, and 25.59 mm, respectively [24,25,28]. In a separate study by El Mekkawi et al., they found that 68.1% of women had CL less than 28 mm, while 31.9% had lengths greater than 28 mm [26]. However, Sharma et al. reported a notably higher estimate, with 50% of women having CL exceeding 30 mm, which is among the highest reported in the studies we reviewed [27]. This variability in CL measurements across different studies may be attributed to differences in the risk profile for cesarean delivery and variations in measurement techniques. All the quoted studies have used TVUS the method of which is standardised. Despite the subjectivity often associated with BS, CL measurements obtained through TVUS are also susceptible to subjectivity. Factors such as bladder voiding, obtaining a longitudinal view of the cervix, identifying the cervical canal and surrounding mucosa, using appropriate magnification, applying correct probe pressure, maintaining the proper duration of the examination, and accurately placing the calipers can all introduce subjectivity into the measurements [10]. To minimize measurement subjectivity, all measurements in our study were performed by a single experienced observer, thus reducing intra-observer variability. In this study, postmaturity (34, 28.3%), preeclampsia (21, 17.5%), and IHCP (17, 14.2%) were the three most common reasons for IOL. The profile of indications for IOL varies substantially across different studies, and with them, the risk of cesarean delivery also varies substantially as each of the risk factors carries different risks for cesarean delivery [22,25,25,27].

In this study, the cesarean rate was 35.8% (43/120). The cesarean rate in this study is thus much higher than the classically cited 20% cesarean rate in the IOL [20]. However, the cesarean rate has shown considerable variability in different studies reported across the literature ranging from 10.7% to 47% (Table 5). This suggests that factors beyond patient profiles play a pivotal role in determining the cesarean rate. The cesarean rate is likely influenced by other variables, such as the availability of intrapartum monitoring facilities at a given medical center and the expertise of the healthcare provider overseeing labor. The proportion of nulliparous women reported across different studies varied from 44.9% to 100%. The mean period of gestation across different studies ranged from 37 to 41.2 weeks. The CS rate across different studies varied from 11% to 47%. Now, in the studies that include nulliparous women exclusively, the CS rate varies from 16.7% to 33.8%, with a mean period of gestation of 38.9 weeks. Studies including a mixed population of nulliparous and multiparous women report CS rates ranging from 11% to 29.6%, with a mean period of gestation of 40.2 weeks. The mean CS rate for induction at the period of gestation less than 40 weeks is 22.94% (range 17%-29.60%), whereas the same for induction at the period of gestation more than 40 weeks is 20.46% (range 13%-33.80%) (Table 5). While formal statistical analysis is not possible due to significant heterogeneity of the reported data across literature, none of these findings correlate with each other to conclude that a higher CS rate has a higher number of nulliparous females, nor does earlier IOL have more chances of failure. Additionally, the indications for the IOL might also impact the cesarean rate, although none of the studies we reviewed explicitly addressed this factor. In light of these considerations, it becomes evident that predicting the likelihood of a CS solely based on patient profiles and characteristics is challenging. This underscores the importance of employing predictive models like the Bishop scoring system and CL measurement to better anticipate the course of labor and make informed clinical decisions regarding cesarean deliveries. In this study, the mean BS of women undergoing CS was significantly lower as compared to that of women undergoing normal delivery, while the mean CL of women undergoing CS was significantly higher as compared to that of women undergoing normal delivery. We did not find any significant association between age, duration of pregnancy, and method of induction with the mode of delivery; however, the cesarean rate was significantly higher in primigravida (23/47, 48.9%) as compared to that in multigravida (20/73, 27.4%). These are similar to the observations made by Cubal et al. who also did not find a significant association of age, body mass index, and gestational age with the mode of delivery but found a significant association of parity with the mode of delivery as observed in this study [22]. As far as parity is concerned, although IOL is targeted toward the achievement of VD, in nullipara/primigravida with an unfavorable cervix undergoing labor induction, the cesarean delivery rate was generally increased and the findings of this study support the same.

When considering both sensitivity and specificity, most studies found CL to be a better predictor than BS (Table 5). While the cutoff value for CL measurements varied across studies, BS cutoff values remained relatively consistent, suggesting a value of less than 5 was suitable (Table 5). In our study, BS showed high sensitivity (83.7%) but low specificity (35.1%), similar to observations in other studies. The PPV of BS remained low across studies, including ours, at only 41.9%. This implies that 58.1% of women identified as at risk of a cesarean delivery based on the BS received unnecessary additional care due to false-positive results, increasing the healthcare burden and costs [22]. In contrast, CL measurements, despite variations in cutoff values, generally exhibited promising combinations of sensitivity and specificity. Most studies indicated a trend of balanced sensitivity and specificity, indicating fewer false positives and false negatives and suggesting good accuracy for CL measurements.

The study's strength lies in its comprehensive and detailed analysis of various factors related to labor induction, including BSs, CL measurements, patient demographics, and indications for induction, providing a thorough understanding of the subject. Additionally, the study's fairly large sample size enhances its statistical power and reliability, allowing for robust conclusions to be drawn from the data. The study is conducted in one of the tertiary care centers of India with a large catchment area mimicking the real-life clinical scenarios. Since all the sonographic measurements were carried out by a single experienced obstetrician, it has high reliability and excludes inter-observer variability.

This study has certain limitations that should be acknowledged. We did not consider maternal body weight, specifically the body mass index (BMI), which is a recognized predictor of cesarean delivery during labor induction. The measurement of CL needs a TUVS machine, which is not easily available in most of the primary or secondary care hospitals of developing countries. The method of labor induction was not standardized in our study, and we could not do a multivariate logistic regression analysis to adjust for these potential confounders.

## Conclusions

The study findings reveal that CL (>26.5 mm) demonstrates a superior predictive ability for the outcome of IOL compared to BS (<5). However, standardization of measurement methods and the derivation of center-specific cutoff values are essential. To enhance the reliability of these findings and develop comprehensive guidelines, further multicentric randomized controlled trials are needed, encompassing various clinical indications for IOL and incorporating diverse induction methods.
